# Protein/CaCO_3_/Chitin Nanofiber Complex Prepared from Crab Shells by Simple Mechanical Treatment and Its Effect on Plant Growth

**DOI:** 10.3390/ijms17101600

**Published:** 2016-09-22

**Authors:** Yihun Fantahun Aklog, Mayumi Egusa, Hironori Kaminaka, Hironori Izawa, Minoru Morimoto, Hiroyuki Saimoto, Shinsuke Ifuku

**Affiliations:** 1 Graduate School of Engineering, Tottori University, 4-101 Koyama-Cho Minami, Tottori 680-8552, Japan; D14T3001B@edu.tottori-u.ac.jp (Y.F.A.); h-izawa@chem.tottori-u.ac.jp (H.I.); morimoto@chem.tottori-u.ac.jp (M.M.); saimoto@chem.tottori-u.ac.jp (H.S.); 2 Faculty of Agriculture, Tottori University, 4-101 Koyama-Cho Minami, Tottori 680-8553, Japan; gonta927@hotmail.com (M.E.); kaminaka@muses.tottori-u.ac.jp (H.K.)

**Keywords:** crab shell, chitin nanofiber, complex, fertilizer

## Abstract

A protein/CaCO_3_/chitin nanofiber complex was prepared from crab shells by a simple mechanical treatment with a high-pressure water-jet (HPWJ) system. The preparation process did not involve chemical treatments, such as removal of protein and calcium carbonate with sodium hydroxide and hydrochloric acid, respectively. Thus, it was economically and environmentally friendly. The nanofibers obtained had uniform width and dispersed homogeneously in water. Nanofibers were characterized in morphology, transparency, and viscosity. Results indicated that the shell was mostly disintegrated into nanofibers at above five cycles of the HPWJ system. The chemical structure of the nanofiber was maintained even after extensive mechanical treatments. Subsequently, the nanofiber complex was found to improve the growth of tomatoes in a hydroponics system, suggesting the mechanical treatments efficiently released minerals into the system. The homogeneous dispersion of the nanofiber complex enabled easier application as a fertilizer compared to the crab shell flakes.

## 1. Introduction

Chitin is a highly abundant carbohydrate polymer occurring primarily in crab shells. Crab shells have a hierarchically-ordered organization [1]. The rigid chitin molecules are aligned in an antiparallel manner to form α-chitin nanofibers with an extended crystalline structure. These nanofibers are covered by a protein layer. The next layer consists of clusters of protein/chitin nanofibers that form a twisted plywood layer, which is gradually rotated about its normal axis. Calcium carbonate, consisting of calcite crystal, is embedded in the small cavity of the helicoidally-shaped structure. In a previous study, we isolated chitin nanofibers from crab shells and prepared them using a simple mechanical treatment [2]. Chitin nanofibers have a characteristic morphology [3], high surface-to-volume ratio [4], high mechanical strength [5,6], and efficient biological properties [7,8,9,10].

The chitin in crab shells is partially preserved in the fishing industry as an intermediate of chitosan and glucosamine, even though the other parts of the shell are disposed of as industrial waste. The chitin is prepared from crab shells by treatment with NaOH and HCl aqueous solutions to remove proteins and calcium carbonate [11]. A major effluent purification process is then required to purify the abundant calcium carbonate and protein residue, and the expense of this process is passed on in the cost of commercial chitin (currently about 5000 Japanese Yen/kg). In a previous study, we investigated methods for skipping the protein removal process to bring down production costs [12]. The protein/chitin nanofiber complex, thus obtained, could reinforce acrylic resin film and increase its mechanical properties. Moreover, the protein layer on the chitin nanofiber behaved as a substrate for the biomineralization of calcium carbonate. On the basis of that study, we hypothesized that it might be possible to convert the chitin in crab shells into nanofiber directly, without removing the protein and calcium carbonate. Simplification of the preparation process would significantly reduce the production cost of nanofiber. Furthermore, eliminating the use of chemicals for purification would render the process more environmentally friendly. In this study, we report on the direct disintegration of crab shells using our proposed method. We then characterize the obtained protein/CaCO_3_/chitin nanofiber complex in detail, and discuss its potential application as a plant fertilizer since it is well known that crab shells show enhancement of plant growth.

## 2. Results and Discussion

### 2.1. Preparation and Characterization of the Protein/CaCO_3_/Chitin Nanofiber Complex

Red snow crab shells were used as a starting material in this study. The contents of the main components in the crab shells (chitin, protein, and calcium carbonate) were estimated by the ninhydrin-hydrindantin protein test and gravimetric analysis; they were approximately 30%, 16%, and 55% as *w*/*w*, respectively, indicating the largest proportion of the red crab shells was calcium carbonate.

**Figure 1:**
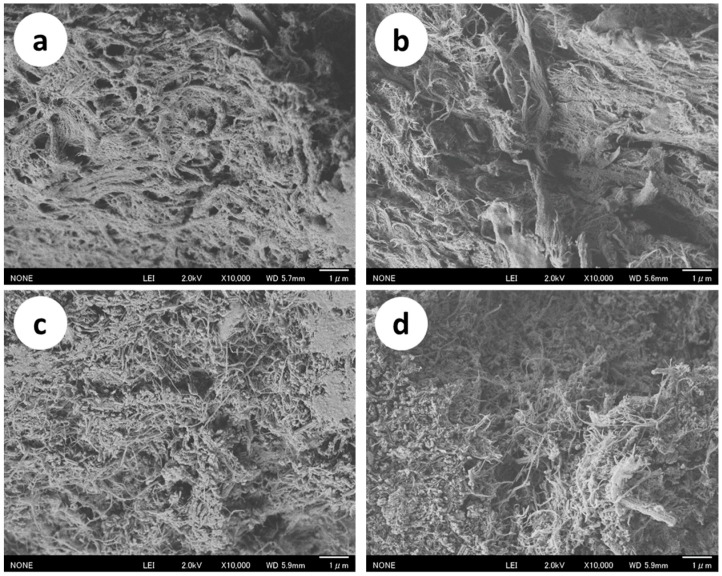

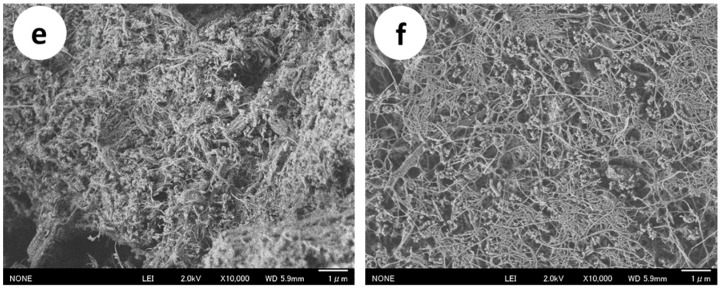
FE-SEM micrographs of crab shells after (**a**) 0; (**b**) 1; (**c**) 5; (**d**) 10; (**e**) 30; and (**f**) 50 passes through the high pressure water jet system. The scale bar length is 1 µm.

Figure 1 shows field emission scanning electron microscope (FE-SEM) images of a crab shell after high-pressure water-jet (HPWJ) treatments with 0, 1, 5, 10, 30, and 50 passes. Before HPWJ treatment (Figure 1a), the crab shell organization consisted of protein/chitin nanofiber bundles with calcium carbonate. After one-pass treatment (Figure 1b), the organization had partially disintegrated, and nano-sized fibers were observed to some extent. The morphological changes in the crab shell after one-pass treatment were due to the strong mechanical load of the HPWJ treatment. When the number of passes increased from 5 to 50, the fiber’s width decreased further due to fibrillation of the protein/chitin nanofiber bundle. Calcium carbonate grains also disintegrated into smaller nanoparticles. Thus, the protein/CaCO_3_/chitin nanofiber complex was successfully obtained from crab shells by HPWJ mechanical treatments. To further characterize the morphology of the nanofiber, calcium carbonate was removed from samples treated with 0, 1, and 5 passes. Calcium carbonate was neutralized by HCl and dissolved in water as CaCl_2_. SEM images revealed that nanofibrillation of the crab shells proceeded by repeated HPWJ treatments and appeared complete at 5 passes (see Figure S1).

**Figure 2:**
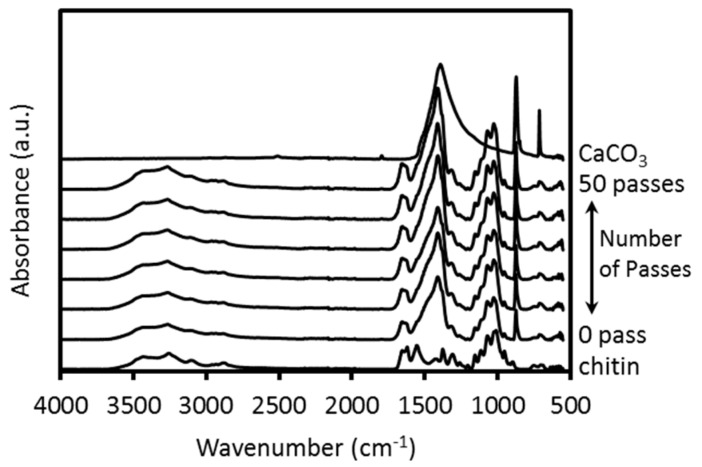
FTIR-ATR spectra of pure chitin, calcium carbonate, and crab shells treated with 0, 1, 5, 10, 30, and 50 passes through a high pressure water jet system.

Figure 2 shows the FTIR-ATR spectra of pure chitin, calcium carbonate, and crab shells treated by the HPWJ system after 0, 1, 5, 10, 30, and 50 passes. All spectra of the mechanically-treated crab shells were in excellent agreement with each other. This suggests that the original chemical structures of the crab shells were maintained after HPWJ mechanical treatments. In particular, the OH stretching band at 3424 cm^−1^, NH stretching band at 3259 cm^−1^, amide I band at 1652 and 1621 cm^−1^, and amide II band at 1554 cm^−1^ of the chitin nanofibers were observed [11]. These absorption peaks are especially characteristic of chitin. The transmittance bands at approximately 870 and 1450 cm^−1^ were derived from calcium carbonate [13]. On the other hand, the peaks corresponding to protein were not observed, due to the weak nature of the protein bands compared to their chitin and CaCO_3_ counterparts.

**Figure 3:**
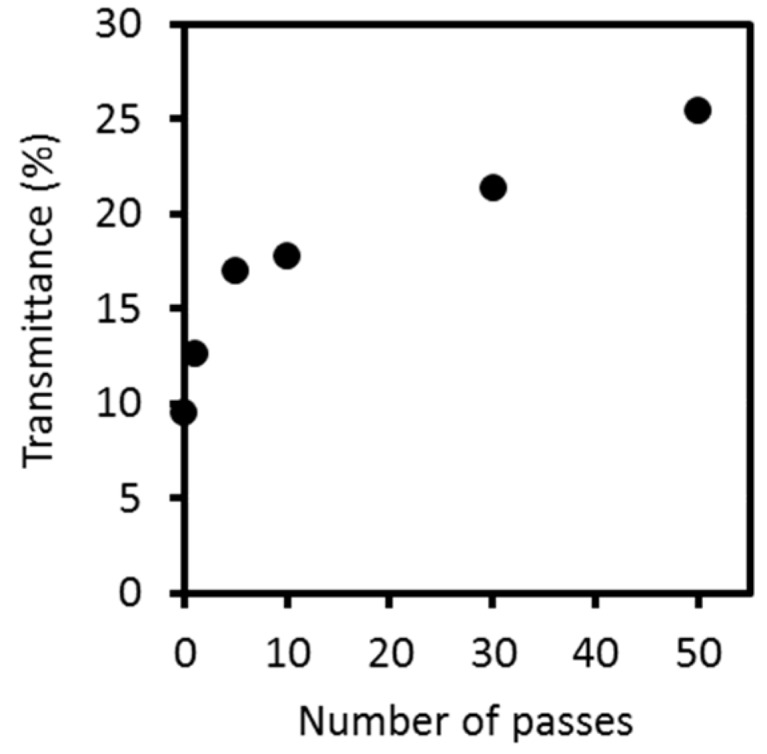
Regular light transmittances of protein/CaCO_3_/chitin nanofiber dispersions at 800 nm as a function of the number of passes.

Figure 3 shows the light transmittances of a 0.1 wt % concentration, mechanically-treated crab shell at 800 nm of water dispersion. Transmittance values were highly reproducible. Transparency is strongly associated with chitin fiber thickness because, when the solid fibers are dispersed at the nano level, the suspension becomes transparent [14]. After HPWJ treatment, the crab shells remained well-dispersed in water for at least one month. Thus, the crab shells were easy to handle and shape into the desired forms. Without HPWJ treatment, the light transmittance of crab shells was only 9.6%. As the number of passes increased, the thickness of the nanofibers decreased; and consequently, the light transmittance of the dispersion increased. At 1 and 5 passes, the transmittance increased steeply to 12.7% and 17.1%, respectively. This shows that the composite nanofibers revealed high morphological change up to 5 passes. Above 10 passes, the transparency did not change significantly, reaching only 25.5% at 50 passes. This trend indicates that—after up to five mechanical treatments—fibers were fibrillated into thinner nanofibers, thus increasing their transparency. However, above 5 passes, the nanofibers were mostly fully fibrillated and further disintegration was difficult—resulting in saturated transparency. These trends agreed with the results of FE-SEM.

**Figure 4:**
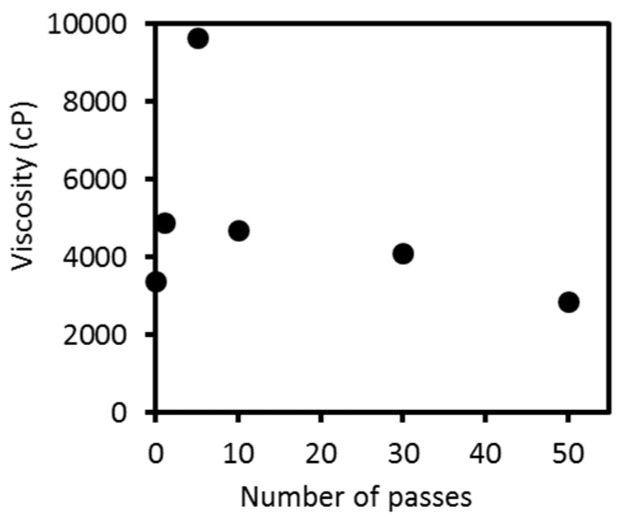
Viscosity of protein/CaCO_3_/chitin nanofiber dispersions as a function of the number of passes.

Figure 4 shows the viscosities of 3.6 wt % mechanically-treated crab shell dispersions as a function of the number of passes. The viscosity values were highly reproducible. The viscosity of the slurry sharply increased from 3380 centipoise (cP) at zero passes to 9633 cP at 5 passes. However, above 10 passes, the viscosity decreased constantly to 2860 cP at 50 passes. The viscosity was strongly affected by nanofiber morphology [15]. The thinner and shorter the chitin nanofibers are, the more frequently the fibers become entangled, which increases the viscosity. The viscosity data indicate that, up to 5 passes, the nanofibers became thinner as the number of passes increased. Above approximately 5 passes, the nanofibrillation was mostly completed, and the fiber length decreased as fibers started to break; thereby lowering the viscosity. Based on the SEM observations, transparency, and viscosity measurement results, it could be inferred that the crab shell composite fibers were properly disintegrated and nanofibrillated at around 5 passes.

### 2.2. Plant Growth Effect of the Protein/CaCO_3_/Chitin Nanofiber Complex

**Figure 5:**
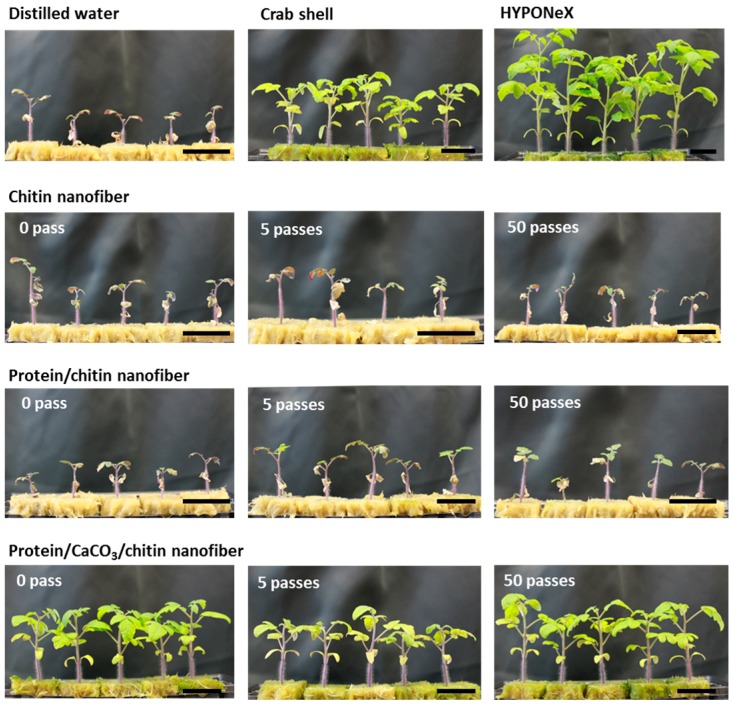
Tomato plants grown hydroponically with nanofibers. Tomato plants were treated with chitin nanofiber, protein/chitin nanofiber, and protein/CaCO_3_/chitin nanofiber at a final concentration of chitin 0.01%. The series of nanofibers were prepared with high pressure water jet treatment with 0, 5, and 50 passes. The photo was taken after the fifth nanofiber treatment. Distilled water was administered as a negative control and commercial nutrition (HYPONeX) as a positive control. Roughly-crushed crab shell powder was treated as a control. The scale bar length is 36 mm.

Tomato plants grown in a hydroponic system were treated with nanofibers once a week. After the fifth nanofiber treatment, the tomato plants treated with protein/CaCO_3_/chitin nanofibers exhibited markedly higher leaf age, stem diameter, and plant height than the controls (Figure 5, Table 1). The effect of the crab shell powder on the tomato plant growth was comparable to that of mineralized chitin-protein composite nanofibers. As noted in Table 1, after the fifth and ninth treatments, the effects of crab shell powder were not significantly different (*p* > 0.05) from those of protein/CaCO_3_/chitin nanofibers with respect to the number of leaves and stem diameter of the plants. However, the effects of the two supplements on the height of the tomatoes after five treatments were significantly different (*p* < 0.05); with the protein/CaCO_3_/chitin nanofibers exhibiting the better effect on tomato growth. The difference was attributed to the effect of mechanical treatment in producing the fast release of minerals deposited in the nanofibers network to the hydroponics system. In addition, mechanically-disintegrated protein/CaCO_3_/chitin nanofibers were easy to apply due to their liquid state, compared to the insoluble, roughly-crushed crab shell powder.

On the other hand, neither protein/chitin nanofibers nor chitin nanofibers had any effect on tomato growth. Tomato plants treated with protein/chitin nanofiber, chitin nanofiber and distilled water (DW) died at the ninth nanofibers treatment; whereas tomato plants treated with protein/CaCO_3_/chitin nanofibers or crab shells showed healthy growth. These results indicated that the protein/CaCO_3_/chitin nanofibers might act as a fertilizer. Plants require not only essential elements—such as nitrogen, phosphorus, and potassium—but also calcium, sulfur, magnesium, and trace minerals for their growth. Although chitin nanofibers and protein/chitin nanofibers contain essential nitrogen, these nanofibers might not contain sufficient amounts of minerals to promote plant growth. Even though the effects of protein/CaCO_3_/chitin nanofibers were not as dramatic as those of the commercial nutrient HYPONeX; after further improvements, nanofibers might show good performance as a fertilizer. The results in Table 1 also revealed that there were no significant differences among the nanofibers treated with a different number of passes of the HPWJ system in terms of tomato growth. Therefore, the 0 pass sample of protein/CaCO_3_/chitin nanofibers, which was roughly crushed by grinder treatment with two passes, might be a cost-effective material for use as a fertilizer for plant growth.

## 3. Materials and Methods

### 3.1. Materials

The raw shells of *Chionoecetes opilio* (red snow crab) and α-chitin powder with a 6.4% deacetylation degree were obtained from Koyo Chemicals. Other chemicals were purchased from Aldrich or Kanto Chemicals and were used as received. Tomato seeds were purchased from the Marutane Seed Co., Ltd., Kyoto, Japan.

### 3.2. Preparation of the Protein/CaCO_3_/Chitin Nanofiber Complex

Raw crab shells were roughly crushed with a domestic blender under wet conditions. The crushed sample was then disintegrated with a grinder (MKCA6-3; Masuko Sanyo Co., Ltd., Kawaguchi, Japan) at 1500 rpm for two cycles. The air bubbles were removed by a super stirrer (Awatori-rentaro, ARE-301, THINKY, Ltd., Tokyo, Japan). The concentration of the total solid was adjusted to 3.6 wt % for a chitin weight percentage of around 1 wt %. The samples were then passed through a high-pressure water-jet system (HPWJ; Star Burst Mini, HJP-25001S, Sugino Machine, Namerikawa, Japan) equipped with a ball collision chamber. The slurry was ejected from a small nozzle with a diameter of 100 µm under high pressure of 200 MPa. This mechanical treatment was repeated for 1, 5, 10, 30, and 50 cycles.

### 3.3. Preparation of the Protein/Chitin Nanofiber Complex and Pure Chitin Nanofibers

Protein/chitin nanofiber complex and pure chitin nanofibers were prepared in order to compare their effects on tomato growth with those of the protein/CaCO_3_/chitin nanofiber complex. The protein/chitin nanofiber complex was prepared using the previously reported procedure, with slight modifications [12]. Briefly, the raw crab shells were crushed with a domestic blender under wet conditions. The crushed sample was treated with 2 M hydrochloric acid for two days at room temperature, with occasional stirring to remove mineral salts (mainly calcium carbonate). The sample was collected by filtration and washed with deionized water until the filtrate became neutral. The sample was then crushed by grinder treatment, and the concentration of the overall solid was adjusted to 1.15 wt %. The ground sample was passed through 5 and 50 cycles of a high-pressure water-jet (HPWJ) system.

For comparison, pure chitin nanofiber was also prepared as described in our previous study, and then modified as follows [16]. Dry pure chitin powder was dispersed in water at 1 wt %. After 1 h of stirring, the dispersion was crushed with a grinder for two cycles. The ground sample was stirred under a vacuum for 1 h to remove air bubbles. Finally, it was mechanically homogenized with 5 and 50 cycles of the HPWJ system.

### 3.4. Characterization

For field emission scanning electron microscopic (FE-SEM) observation, the disintegrated samples were diluted with ethanol and dried in an oven. The dried samples were coated with an approximately 2 nm layers of platinum, using an ion sputter coater, and were observed by an FE-SEM (JSM-6700F; JEOL, Tokyo, Japan) operating at 2.0 kV. Infrared spectra of the samples was recorded with an FT-IR spectrophotometer (Spectrum 65; Perkin-Elmer Japan, Yokohama, Japan) equipped with an ATR attachment (diamond/ZnSe crystal) with four scans at a resolution of 4 cm^−1^. The light transmittances of the protein/mineral/chitin nanofiber complex dispersion were measured using a UV-Vis spectrophotometer (V550; JASCO, Tokyo, Japan). The viscosities of the complex slurries were measured with a Brookfield digital viscometer DV-E using the LV-4 spindle (Brookfield Engineering Laboratories, Middleboro, MA, USA) at 30 °C. The protein, calcium carbonate, and chitin contents of the original crab shells were estimated by means of a ninhydrin-hyrindantin protein test [17] and gravimetric analysis. Calcium carbonate and protein were separated from the crab shells by conventional 1 M HCl and 1 M KOH treatments, respectively.

### 3.5. Plant Material

Tomato (*Solanum lycopersicum*) cv. Chibikko (Marutane Seed) was grown hydroponically with rockwool grow cubes (AO 36/40; Grodan). Three weeks after seeding, the tomato plants were treated with a series of nanofibers, which contained chitin at a final concentration of 0.01% to each rockwool cube. The number of true leaves, the stem diameter (at cotyledon node), and the plant height (stems length above the cotyledon) were measured after the fifth and ninth nanofiber treatment. The plants were grown under environmentally-controlled conditions with 14 h of light/10 h of dark cycles at 25 °C, and treated with nanofibers once a week. Distilled water (DW) was used as a negative control, and commercial nutrition HYPONeX (N-P-K = 6-10-5; Hyponex Japan, Osaka, Japan) at a concentration of 0.001% was used as a positive control. Roughly-crushed crab shell powder was treated as a control.

## 4. Conclusions

A protein/CaCO_3_/chitin nanofiber complex was prepared from crab shells using a simple mechanical treatment. Because this nanofiber preparation process did not include the conventional protein and calcium carbonate removal processes, it could bring down the production cost and environmental load. The resulting protein/CaCO_3_/chitin nanofiber would be available as a fertilizer to improve plant growth in hydroponic systems. Indeed, the mineral part of the nanofiber complex was found to be vital for plant growth and maturation. Finally, the novel nanofiber complex could be prepared at a low cost through an eco-friendly process. Thus, the results of this close analysis of the unique nanofiber-complex characteristics could lead to more effective utilization of crab shell waste.

## Figures and Tables

**Table 1:** The effect of protein/CaCO_3_/chitin nanofibers on the growth of tomato plants.

Day after Treatment	Treatment	Number of Passes	Number of Leaves	Stem Diameter (mm)	Plant Height (cm)
5 weeks	Distilled water	-	3.2 ± 0.1 ^a^	1.4 ± 0.0 ^a^	0.9 ± 0.1 ^a^
	HYPONeX	-	8.3 ± 0.2 ^b^	3.0 ± 0.2 ^b^	11.6 ± 0.8 ^b^
	Crab shell	-	6.0 ± 0.3 ^c^	2.0 ± 0.1 ^c^	4.3 ± 0.2 ^c,d,e^
	Protein/CaCO_3_/chitin nanofiber	0	6.3 ± 0.2 ^c^	2.2 ± 0.1 ^c^	6.8 ± 1.9 ^c^
		5	5.8 ± 0.3 ^c^	2.0 ± 0.1 ^c^	4.4 ± 0.4 ^c,d^
		50	6.7 ± 0.2 ^c^	2.2 ± 0.1 ^c^	5.0 ± 0.1 ^c^
	Protein/chitin nanofiber	0	3.1 ± 0.2 ^a^	1.4 ± 0.0 ^a^	1.0 ± 0.1 ^a^
		5	3.5 ± 0.2 ^a^	1.5 ± 0.0 ^a^	1.4 ± 0.1 ^a,d,e^
		50	3.6 ± 0.2 ^a^	1.5 ± 0.0 ^a^	1.2 ± 0.1 ^a,e^
	Chitin nanofiber	0	3.2 ± 0.1 ^a^	1.4 ± 0.0 ^a^	1.0 ± 0.1 ^a^
		5	3.0 ± 0.3 ^a^	1.4 ± 0.0 ^a^	1.0 ± 0.1 ^a^
		50	3.0 ± 0.1 ^a^	1.4 ± 0.0 ^a^	0.9 ± 0.0 ^a^
9 weeks	Distilled water	-	ND		
	HYPONeX	-	9.7 ± 0.4 ^a^	3.3 ± 0.1 ^a^	16.6 ± 0.5 ^a^
	Crab shell	-	8.4 ± 0.5 ^a^	2.1 ± 0.1 ^b^	7.0 ± 0.9 ^b^
	Protein/CaCO_3_/chitin nanofiber	0	9.1 ± 0.3 ^a^	2.2 ± 0.1 ^b^	9.1 ± 0.6 ^b^
		5	8.6 ± 0.3 ^a^	2.2 ± 0.1 ^b^	7.8 ± 0.6 ^b^
		50	9.2 ± 0.3 ^a^	2.3 ± 0.1 ^b^	9.3 ± 0.5 ^b^
	Protein/chitin nanofiber	0	ND		
		5	ND		
		50	ND		
	Chitin nanofiber	0	ND		
		5	ND		
		50	ND		

Data represents the mean of four independent experiments and error bars. Means with the same letter (a–e) are not significantly different according to Tukey’s test (*p* < 0.05) in each week. ND indicates dead plant.

## Supplementary Materials

Supplementary materials can be found at www.mdpi.com/1422-0067/17/10/1600/s1.
