# Supplementary material for: Protein/CaCO3/Chitin Nanofiber Complex Prepared from Crab Shells by Simple Mechanical Treatment and Its Effect on Plant Growth
Source: Int J Mol Sci. 2016 Sep 22;17(10):1600. doi: 10.3390/ijms17101600 (PMC5085633; doi:10.3390/ijms17101600)
Supplement: Supplementary file 1 [file ijms-17-01600-s001.pdf]

Supplementary Materials: Protein/$CaCO_3$/Chitin Nanofiber Complex Prepared from Crab Shells by Simple Mechanical Treatment and Its Effect on Plant Growth

Yihun Fantahun Aklog, Mayumi Egusa, Hironori Kaminaka, Hironori Izawa, Minoru Morimoto, Hiroyuki Saimoto and Shinsuke Ifuku

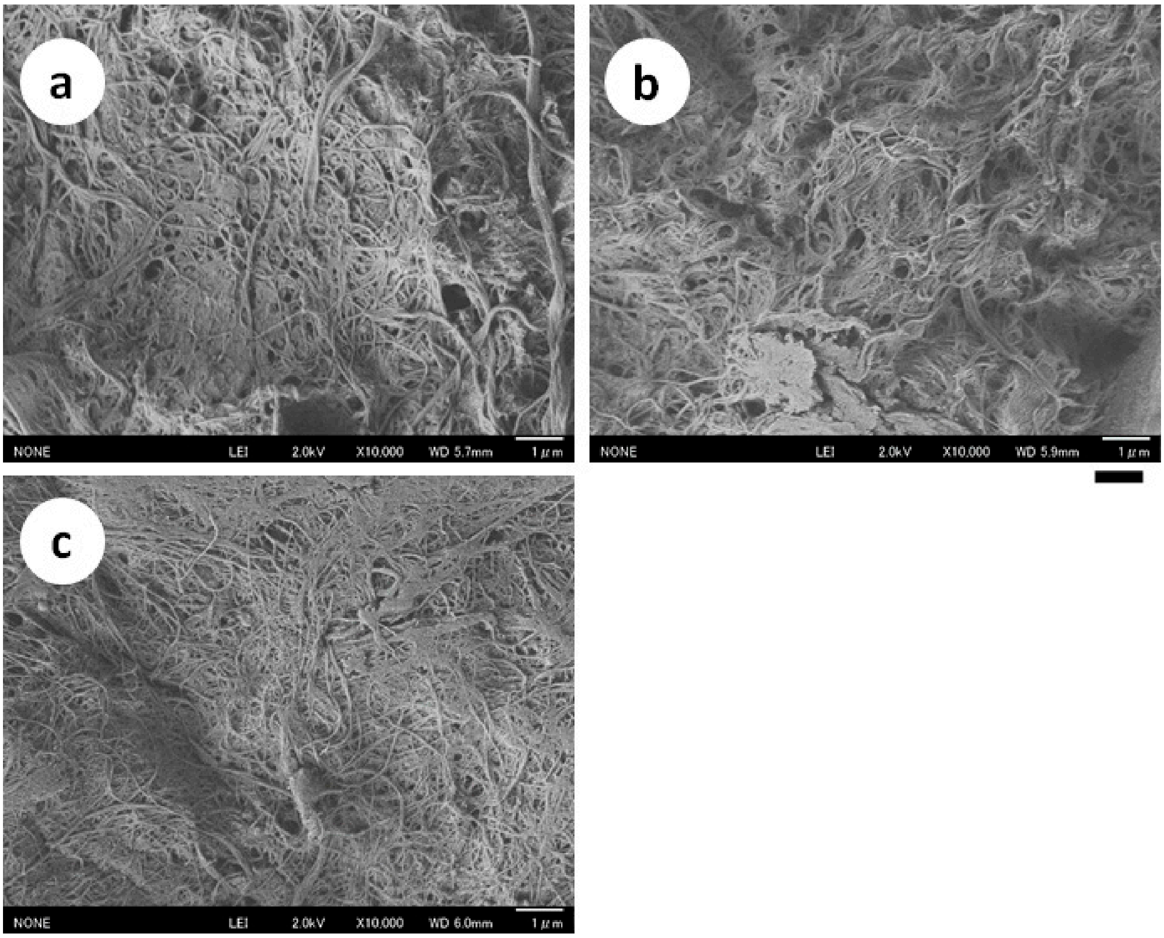

Figure S1. FE-SEM micrographs of crab shells with (**a**) 0-; (**b**) 1-; and (**c**) 5-cycle treatments through the high pressure water jet system after removal of protein and $CaCO_3$. The scale bar length is 1 μm.
